# Dyspnoea and restrictive lung disease due to mediastinal and pleural lipomatosis in morbid obesity

**DOI:** 10.1002/rcr2.421

**Published:** 2019-04-09

**Authors:** Jen Yuh Lim, Kim A. McAnulty, Catherina L. Chang

**Affiliations:** ^1^ Department of Respiratory Medicine Waikato Hospital Hamilton New Zealand; ^2^ Department of Radiology Waikato Hospital Hamilton New Zealand

**Keywords:** Dyspnoea, lipomatosis, mediastinal neoplasms, obesity, pleural neoplasms

## Abstract

Dyspnoea in obese patients can be multifactorial and complex. Mediastinal and pleural lipomatosis can be associated with obesity and is usually considered asymptomatic and benign. We report an obese 39‐year‐old man who presented with progressive dyspnoea, where in addition to obstructive sleep apnoea and obesity hypoventilation syndrome, was found to have massive mediastinal and pleural lipomatosis causing restrictive lung disease. Pleural lipomatosis are generally slow growing so conservative management is recommended. However, complications such as haemorrhage and compression of adjoining organs can occur in pleural lipomas, so surgical excision can be considered in some instances.

## Introduction

Exertional dyspnoea in obese individual are common and may be due to a number of factors. Obesity contributes to reduced resting and exercise lung volumes; reduced lung and chest wall compliance; and increased oxygen cost of breathing. It is associated with cardio‐metabolic disease which may limit exercise. Obese individuals also often have reduced conditioning. Mediastinal lipomatosis is rare and most often seen in patients with Cushing's syndrome and in chronic steroid use, although obesity is also an association. Mediastinal and pleural lipomatosis is usually considered benign, causing little symptoms 1, 2. Literature is sparse regarding the contribution of mediastinal or pleural lipomatosis to dyspnoea. We present a case of large volume mediastinal and pleural lipomatosis causing significant dyspnoea in a morbidly obese individual.

## Case Report

A 39‐year‐old New Zealand man of European descent initially presented with syncopal events in the context of excessive daytime sleepiness and exertional dyspnoea. He was a never‐smoker and had a community clinical diagnosis of asthma with no objective evidence of bronchial hypersensitivity. Examination revealed morbid obesity (body mass index 48.8 kg/m^2^) and retrognathia. Epworth sleepiness score was 23/24 and arterial blood gas showed awake hypercapnia consistent with obesity hypoventilation syndrome. Overnight sleep study confirmed severe sleep disordered breathing (apnoea‐hypopnoea index 143/h, lowest saturations of 61%). He was issued home continuous positive airway pressure treatment, however was not adherent to therapy. Subsequently he presented to hospital five times over six months with increasing dyspnoea. While he was unable to perform acceptable spirometric manoeuvres, carbon dioxide transfer factor was 5.1 mmol/kPa/min (62% predicted). Peak expiratory flow rates during admission showed minimal reversibility (350 mL, 70% predicted) and symptoms persisted despite treatment with long‐ and short‐acting bronchodilators. Transthoracic echocardiogram was attempted but technically limited due to the patient's body habitus. It showed normal concentric left ventricular wall thickening, with both ventricles of normal size and systolic function, and no valvular pathology seen.

**Figure 1:**
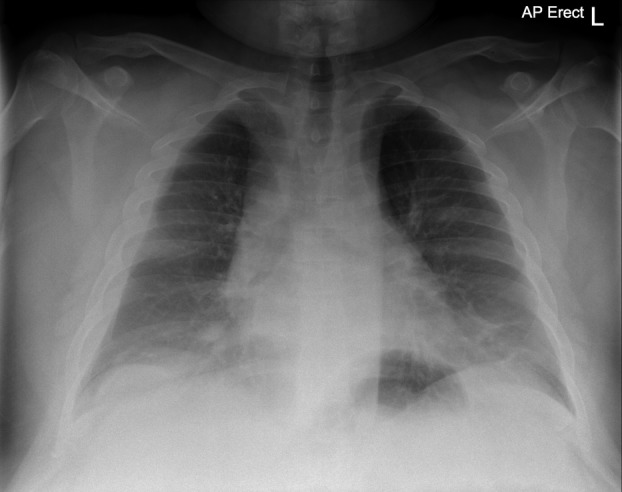
Chest X‐ray showing moderate bilateral widening of the superior mediastinum with cardiomegaly and visible pleural fat laterally.

**Figure 2:**
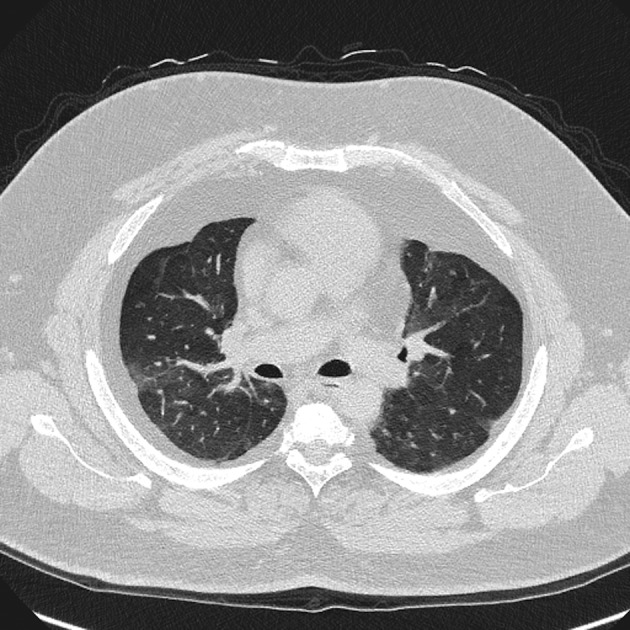
Computed tomography chest showing extensive mediastinal and pleural fat accumulation.

The patient underwent a high‐resolution computed tomography (CT) to exclude abnormalities of lung parenchyma contributing to dyspnoea. The predominant abnormality was large volumes of mediastinal and pleural fat. The pleural fat was predominantly based around the upper lobes with obtuse margins without any significant lobulation and had an average density of −110 Hounsfield units, consistent with lipomatous tissue (Figs. 1, 2). Following multi‐disciplinary team discussion, review of images, and all available results, a diagnosis of exertional dyspnoea secondary to obesity with significant pulmonary restriction due to pleural and mediastinal lipomatosis was made. The recommendation was adherence to CPAP and weight loss.

## Discussion

Dyspnoea is a very common symptom in obesity with multiple potential mechanisms. Characteristically, obese individuals have reduced end‐expiratory lung volume, increased work of inspiratory muscles, reduced lung and chest wall compliance, and increased oxygen cost of breathing. Obesity is also associated with cardio‐metabolic disease which may limit exercise tolerance. In addition to reduced conditioning, there is also evidence of abnormal central respiratory gating in obese individuals. However, direct large volume deposition of lipomatous tissue in the pleural space and mediastinum causing restrictive lung disease is uncommon.

Lipomas are the most frequently observed benign tumour in adults and have been found at mediastinal, pleural, bronchial, and pulmonary levels intrathoracically 3. Literature on the subject is sparse and these are usually described as stable, slowly progressing tumours 3. While mediastinal lipomatosis is relatively common, simultaneous occurrence of both mediastinal and pleural lipomatosis is rare. We can only find one other case of mediastinal lipomatosis in association with pleural lipomatosis in the literature 4.

The majority of patients with mediastinal lipomatosis are asymptomatic, although some can present with thoracic pain, dyspnoea, cough, dysphonia, and supraventricular tachycardia 5. Pleural lipomas, on the other hand, have been reported to cause complications such as haemorrhage and adjoining organ compression 3. Extrapleural fat occurs typically along the chest wall along the posterolateral aspects of the fourth to eighth ribs bilaterally. On plain film, the resulting soft‐tissue shadow that is produced can be confused with pleural thickening or pleural plaques. Deposition of fat into multiple organs (extra‐pleural, mediastinal, liver, bowel wall) has been recognized to be associated with obesity and commonly seen 6.

CT plays an important role in diagnosing mediastinal and pleural lipomatosis and should be the image modality of choice 7. This helps differentiate between benign and malignant pleural disease and determine the extent and location of disease 7. They usually involve the anterior mediastinum, with a density comparable with subcutaneous fat between −50 and −150 Hounsfield units 3. Bariatric patients’ access to CT is limited by their ability to physically fit into the CT scanner. In our institution this patient was imaged with Philips iQon CT: table load limit of 295 kg, gantry aperture of 70 cm, scan field of view is 50 cm, maximum tube voltage of 140 kVp, and iDose iterative reconstruction was used to optimize images and reduce radiation dose. This CT scanner has been designed to accommodate bariatric patients.

Conservative treatment with weight reduction is the usual first‐line recommendation. If there is endogenous steroid use, cessation can lead to improvement. Surgical excision can be considered in rare occasions, for example if there is direct organ compression 3. Surgical treatment is generally definitive with a low incidence of recurrence. Transformation of pleural lipomatosis into sarcoma is rare 4.

Clinicians should consider mediastinal and pleural lipomatosis as a potential diagnosis when working up obese patients with exertional dyspnoea, especially where other causes of dyspnoea have been excluded. This is especially relevant as the prevalence of overweight and obesity has reached epidemic levels over the last few decades, so we may expect to see similar cases with time.

### Disclosure Statement

Appropriate written informed consent was obtained for publication of this case report and accompanying images.
